# Identification and characteristics of distressed patients with coronary heart disease and insufficiently controlled medical risk factors: baseline findings and sex differences from the multicenter TEACH trial

**DOI:** 10.3389/fpsyt.2025.1494839

**Published:** 2025-01-31

**Authors:** Christoph Herrmann-Lingen, Monika Sadlonova, Ingrid Becker, Kristina Bersch, Franziska Geiser, Martin Hellmich, Ingrid Kindermann, Matthias Michal, Mariel Nöhre, Astrid Petersmann, Rolf Wachter, Birgit Herbeck Belnap, Christian Albus

**Affiliations:** ^1^ Department of Psychosomatic Medicine and Psychotherapy, University Medical Center Göttingen, Göttingen, Germany; ^2^ German Center for Cardiovascular Research (DZHK), partner site Lower Saxony, Göttingen, Germany; ^3^ Department of Cardiothoracic and Vascular Surgery, University Medical Center Göttingen, Göttingen, Germany; ^4^ Department of Geriatrics, University Medical Center Göttingen, Göttingen, Germany; ^5^ Department of Psychiatry, Massachusetts General Hospital, Boston, MA, United States; ^6^ Institute of Medical Statistics and Computational Biology, Faculty of Medicine and University Hospital Cologne, University of Cologne, Köln, Germany; ^7^ Clinical Trials Unit, University Medical Center Göttingen, Göttingen, Germany; ^8^ Department of Psychosomatic Medicine and Psychotherapy, University Hospital of Bonn, University of Bonn, Bonn, Germany; ^9^ Department of Internal Medicine III, Saarland University Medical Center and Saarland University Faculty of Medicine, Homburg, Germany; ^10^ Department of Psychosomatic Medicine and Psychotherapy, University Medical Center Mainz, Mainz, Germany; ^11^ Department of Psychosomatic Medicine and Psychotherapy, Hannover Medical School, Hannover, Germany; ^12^ Institute of Clinical Chemistry and Laboratory Medicine, University Medicine Oldenburg, Oldenburg, Germany; ^13^ Institute of Clinical Chemistry and Laboratory Medicine, University Medicine Greifswald, Greifswald, Germany; ^14^ Department of Cardiology, University Hospital of Leipzig, Leipzig, Germany; ^15^ Central German Heart Alliance, Leipzig, Germany; ^16^ Center for Behavioral Health and Smart Technology, University of Pittsburgh Medical School, Pittsburgh, PA, United States; ^17^ Department of Psychosomatic Medicine and Psychotherapy, University Hospital of Cologne, Köln, Germany

**Keywords:** coronary heart disease, cardiovascular risk factors, psychological distress, blended collaborative care, sex differences

## Abstract

**Introduction:**

Medical risk factors and psychological distress are important targets for secondary prevention of coronary heart disease (CHD). The multicenter randomized controlled TEACH study is the first trial testing a blended collaborative care (BCC) intervention vs. usual care in a cohort of only patients with CHD. The current manuscript analyzes the availability of distressed CHD patients for a BCC intervention trial and the baseline risk profile of the randomized cohort, especially focusing on sex differences.

**Methods:**

Hospitalized CHD patients with positive HADS and/or PSS-4 screening were rescreened three months later and those still distressed were offered participation in the RCT if they had insufficiently controlled medical risk factors (smoking, physical inactivity, elevated blood pressure, LDL cholesterol, and/or HbA1c). The current manuscript describes the TEACH screening process and presents baseline data of the randomized cohort.

**Results:**

Of 2,785 screened patients, 457 patients with persistent distress and insufficiently controlled risk factors were randomized. Older age and lower distress but not sex independently predicted dropout before randomization. In the randomized cohort (mean age 62.9 ± 9.5 years, 77.4% men), women were older than men (p=0.025), more likely to be retired (52.4% vs. 38.6%; p=0.012) and to live without a partner (48.6% vs. 24.8%, p<0.001). Compared to men, they had lower diastolic blood pressure (p=0.003) but higher rates of physical inactivity (56.0% vs. 41.8%; p=0.012) and positive family history of premature atherosclerotic disease (45.7% vs. 29.8%; p=0.009). They also had a lower rate of previous coronary bypass surgery (21.0% vs. 39.2%, p<0.001). A mental disorder had been diagnosed in 54% of all randomized patients and 42% had previously received mental health treatment, both reported substantially more frequently by women than men (both p<0.001). Satisfaction with care before the trial did not differ by sex but was far lower for psychosocial care than for treatment of heart disease (p<0.001).

**Discussion:**

TEACH enrolled a patient sample with persisting distress and a typical risk factor profile. Women differed from men in relevant aspects of their RF profiles and mental health and should receive special attention in future analyses and treatment planning for patients with CHD.

**Clinical Trial Registration:**

German Clinical Trials Register, https://drks.de/search/de/trial/DRKS00020824, identifier DRKS00020824.

## Graphical Abstract

Sex differences in biopsychosocial risks of distressed CHD patients. Physical, psychological, and social risks contribute to poor quality of life. The spider diagram shows significant sex differences in aspects of biopsychosocial risk as observed at baseline in the randomized sample of the multicenter TEACH trial (n=457). White area: women; gray area: men. Scale applies to all variables with their respective units: DBP [mmHg], age [years], others [%].
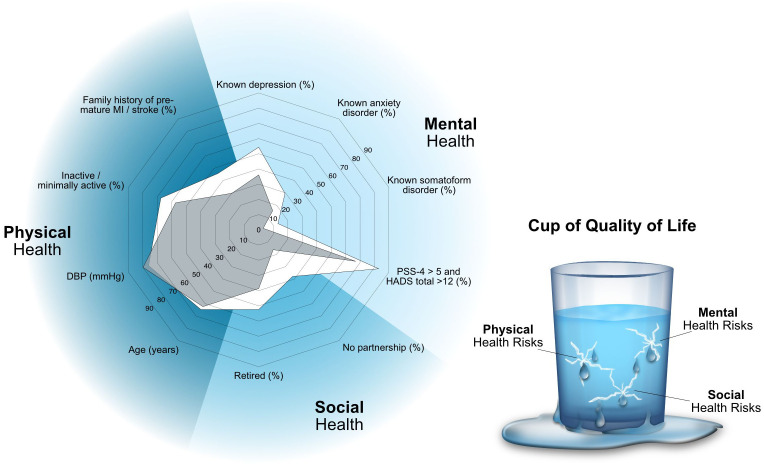

## Introduction

Psychological distress, e.g. anxiety, depression or high perceived stress and medical risk factors such as hypertension, hyperlipidemia, diabetes, smoking and physical inactivity are important targets to address in secondary prevention of coronary heart disease (CHD) (1). While effective drug treatments are available for controlling blood pressure, lipid and glucose levels, their effectiveness is limited by poor adherence (2). Most psychological intervention studies in this area only targeted individual risk factors, such as depression (3–5), stress regulation (6, 7) or physical inactivity (8) without specifically addressing other medical risk factors or treatment adherence. As CHD prognosis depends on medical and psychological risk factors, best effects should be expected from combined treatments for psychological and medical risk factors. However, although recommended by current guidelines (1), integrated treatments simultaneously addressing psychological and medical risk in CHD patients are scarce.

Blended collaborative care (BCC), combining patient-centered support for both, physical and mental health problems, has been shown in a first study to improve psychosocial and medical risk in patients with diabetes/CHD (9). In that study, trained non-physician care managers stayed in regular, proactive telephone-based contact with primary care patients to support them in dealing with depressive symptoms and implementing a healthy lifestyle in their daily routine. During case review meetings, the care managers presented their patients to an interdisciplinary specialist team who made evidence-based treatment recommendations while the patients’ primary care providers remained responsible for their treatment.

Based on the positive results, BCC has been tested under routine conditions in the USA (10) but aside from a small German feasibility study (11), to date no study has tested BCC in any patient group in Europe, and to our knowledge no study has ever tested BCC in a cohort of only CHD patients. Therefore, our randomized controlled TEACH trial (Efficacy of team−based collaborative care for distressed patients in secondary prevention of chronic coronary heart disease) examines the effects of a 12-month telephone-based BCC intervention complementing usual care (UC) in CHD patients with elevated distress and at least one insufficiently controlled medical risk factor vs. UC alone in seven German health centers (12). The trial intervention is delivered by trained non-physician care managers. In a first step, individual health goals are agreed upon between patients and care managers and goal attainment is monitored and supported by regular telephone contacts. Care managers hold regular case review meetings with a specialist team consisting of a psychologist and medical specialists in cardiology and psychosomatic medicine, in order to assure guideline-based treatment recommendations. However, the patient’s treating physician (e.g., general practitioner or cardiologist) remains in charge of making any medical treatment adjustments or ordering additional tests. For details see (12). The main outcome is disease-specific quality of life and secondary outcomes include cardiovascular risk factors, medical events, psychological distress, and treatment satisfaction.

Results from some previous research suggest that responses to psychosocial treatments tend to differ by sex [e.g., (13, 14)] and such treatments should therefore specifically address different needs of men and women [e.g., (15, 16)]. While BCC is strongly based on individual patient preferences and treatment needs it is important to sensitize care managers for typical sex differences in problem areas and care needs, so that they can specifically enquire for such issues during individual intervention planning and delivery.

The current manuscript addresses two main research questions:

First, it examines the effects of the stepwise screening and enrollment algorithm developed for TEACH. In order for BCC to be effective in a CHD sample, identification and selection of a patient sample with both, high psychological distress and modifiable cardiovascular risk factors is essential. A stepwise screening approach was chosen to exclude patients with only transient distress related to hospitalization (16). In addition, many patients in Germany receive sufficient benefit from cardiac rehabilitation which is frequently offered for 3-4 weeks after an acute cardiac event (12), so they may be sufficiently treated with UC thereafter. Screening for insufficiently controlled risk factors three months after hospitalization is also likely to detect patients with ongoing problems in risk factor control. Furthermore, it was of interest which factors can be identified to influence dropout before randomization, in order to increase awareness of the unavoidable selection biases associated with any criteria-based enrollment procedure.

Second, we provide detailed descriptive baseline data of the randomized patient cohort with a particular focus on sex differences in sociodemographic, medical and psychological baseline data. This information will be helpful for identifying research questions to study in posthoc analyses of the TEACH dataset and for planning future BCC or other psychosocial intervention trials in CHD patients.

Finally, the current analysis looks at satisfaction with the treatment that our CHD patients received before enrollment in the RCT assuming that treatment satisfaction will show needs for improvement.

## Methods

TEACH is a multicenter, randomized controlled, confirmatory, interventional trial conducted in accordance with the Declaration of Helsinki. It has been approved by all ethics committees at the seven participating University Hospitals in Göttingen, Cologne, Bonn, Hannover, Homburg, Leipzig, and Mainz. Between August 2020 and April 2023, hospitalized patients aged 18-85 years with CHD were screened for eligibility. To identify patients with persistent psychological distress, we applied a stepwise consent, screening, and enrollment procedure across two timepoints.

### Eligibility screening

#### Screening 1

At participating centers, study personnel approached patients with a suspected diagnosis of CHD who were hospitalized in departments of cardiology or cardiac surgery and gave their verbal consent to be contacted: Patients were given a brief description of the study, and signed the first written informed consent if they were interested in participating. All patients were specifically informed that the RCT tested an intervention to alleviate distress and cardiovascular risk burden. Those who met all inclusion criteria (see (12) and **Supplementary Table 1**) were then screened for psychological distress with the Hospital Anxiety and Depression Scale (HADS > 12) (17, 18) and the Perceived Stress Scale 4 (PSS-4 >5) (19). For details see section on assessments below. At those sites where routine distress screening had already been established (Leipzig, Department of Cardiothoracic and Vascular Surgery in Göttingen), only patients with elevated distress levels were contacted. Furthermore, patients could also be included via self-referral, if they had documented CHD and had been hospitalized within the last 6 months.

In patients who were distressed at this first screening possible inclusion and exclusion criteria were examined from patient records and discharge letters. This included verification of CHD diagnoses based on the criteria shown in **Supplementary Table 1**. Eligible patients received a brief TEACH study brochure, and the hospital physicians were informed about their elevated HADS/PSS-4 scores.

All data were entered into a Good Clinical Practice (GCP) compliant database (secuTrial^®^), specifically programmed and configured for the TEACH trial and regularly monitored for data quality.

#### Screening 2

Study recruiters contacted via telephone all eligible patients from Screening 1 for a confirmative second study screening (Screening 2) three months later. Only in those who reported elevated HADS and/or PSS-4 scores we assessed the presence of the following modifiable CHD risk factors based on European Society of Cardiology (ESC) cardiovascular prevention guidelines in force at the time of study inception (20):

1. Blood pressure ≥ 140/90 mmHg;
2. LDL cholesterol ≥ 70 mg/dl;
3. current smoking;
4. HbA1c ≥ 7.0%;
5. physical inactivity defined as < 150 min. of moderate or < 75 min of vigorous physical activity per week.

We then computed a composite medical risk factor score as a sum (0-5) of the above mentioned insufficiently controlled CHD risk factors (21). Patients who had a medical risk score ≥ 1 and continued to meet all other eligibility criteria were invited to participate in the RCT.

#### Enrollment in RCT

After Screening 2 we administered in person our baseline assessment to all eligible patients who signed a second written informed consent, and after completion patients were randomized in a 1:1 ratio to either UC or BCC. After randomization, study personnel informed patients in the enrolling center and their physician (primary care provider and/or cardiologist) via a letter of their group assignment. Patients randomized to the intervention group then received UC plus 12-months of phone-based BCC (12).

### Assessments

The HADS (17) is a self-rating instrument for assessing anxiety and depressive symptoms on 14 four-step Likert-scaled items, 7 of which address anxiety (sample item: “Worrying thought go through my mind”) and 7 focus on depressive mood [sample item: “I look forward with enjoyment to things” (inverted)]. Subscale scores range from 0 to 21, with higher scores indicating higher symptom load. The total score (range 0-42) can be used as a global measure of distress (18). The German HADS version has been validated in large samples of cardiac patients, mainly with CHD and has shown to predict adverse prognosis in this patient group (18). German population norms are also available (18). Its reliability is comparable to the English version with Cronbach’s alpha reported as 0.80 for the anxiety and 0.81 for the depression subscale (18). Cronbach’s alpha for the total score was 0.87 in the current sample. A cuff-off score of > 12 has been used in previous research [see e.g. (22)] and was chosen here in order to identify even mild levels of distress.

The PSS-4 (19) is a brief assessment of perceived stress consisting of 4 five-step Likert scaled items (range 0-16). The German items were drawn from the published German version of the PSS-10. In the German validation study (23), Cronbach’s alpha of the PSS-10 was 0.84. Due to the smaller item number, alpha for the PSS-4 was 0.65 in the current sample. Similar values have been reported for the English PSS-10 and PSS-4 (24). PSS-4 values > 5 have been found prognostically relevant in CHD patients (25).

Patients were considered distressed if they scored above the cut-off on either (or both) of the two scales (HADS > 12 and/or PSS-4 > 5).

At baseline, physical inactivity we assessed with the 7-item International Physical Activity Questionnaire [IPAQ-7; (26)], a brief 7-Item instrument reporting minutes, hours and days of strenuous or moderate physical activity and sedentary time over a period of 7 days. Social demographics, medical history, and clinical characteristics were obtained from patients' self report, medical documents, and clinical examination. Three *ad-hoc* items with five-point Likert scales asked for satisfaction with medical treatment before study enrollment, separately for medical care in general, treatment for distress, and treatment for heart disease.

### Blood samples

At baseline, venous blood samples were collected in 2.7 ml K3 EDTA tubes from the antecubital vein of the study participants after 15 min of rest in a prone position. EDTA samples were centrifuged, and both, supernatants and red blood cells were subsequently stored at −80°C until further analyses by a specialized laboratory. After internal quality control, LDL-cholesterol was determined using a homogeneous enzymatic color test (Roche Diagnostics, Mannheim, Germany). HbA1c was measured from thawed erythrocytes using a high-performance liquid chromatography (HPLC) with a non-porous cation exchanger employing the potential difference (HLC-723G11, Tosoh Bioscience, Griesheim, Germany).

### Statistical analysis

Descriptive data are reported for the steps of the screening and enrollment process and for baseline data of the randomized sample. Multivariable baseline predictors of dropout before randomization in patients with positive first screening were calculated using logistic regression analysis. In the randomized study sample, continuous variables are reported as mean values (M) and standard deviations (SD). Categorial variables are expressed as numbers and percentages (%). For dichotomous self-report variables such as history of diabetes, hypertension or depression the numbers and percentages of positive answers are presented. If patients weren’t sure about the existence of a particular condition (which happened in less than 3% of cases each), such uncertain answers were counted as “no”. Sensitivity analyses counting "don't know" answers as missing yielded similar results. Differences between men and women in categorical variables were tested with Pearson’s chi-squared tests. The differences in continuous variables were tested with Welch-corrected two-sample *t*-test. Missing values for objective measures such as laboratory values or ejection fraction were excluded from bivariate analyses on a pairwise level. As missingness could be considered as completely at random, complete data were available for all such variables in at least 95% of cases, and sex differences were far from significant no missing value replacement was applied. A significance level of all analyses was set at 0.05 two-sided. All analyses were performed using the software SPSS version 29 (IBM Inc., Armonk, NY, USA) and SAS 9.4 (SAS Institute Inc., Cary, NC, USA)

## Results

### Results of the stepwise screening procedure

**Figure 1:**
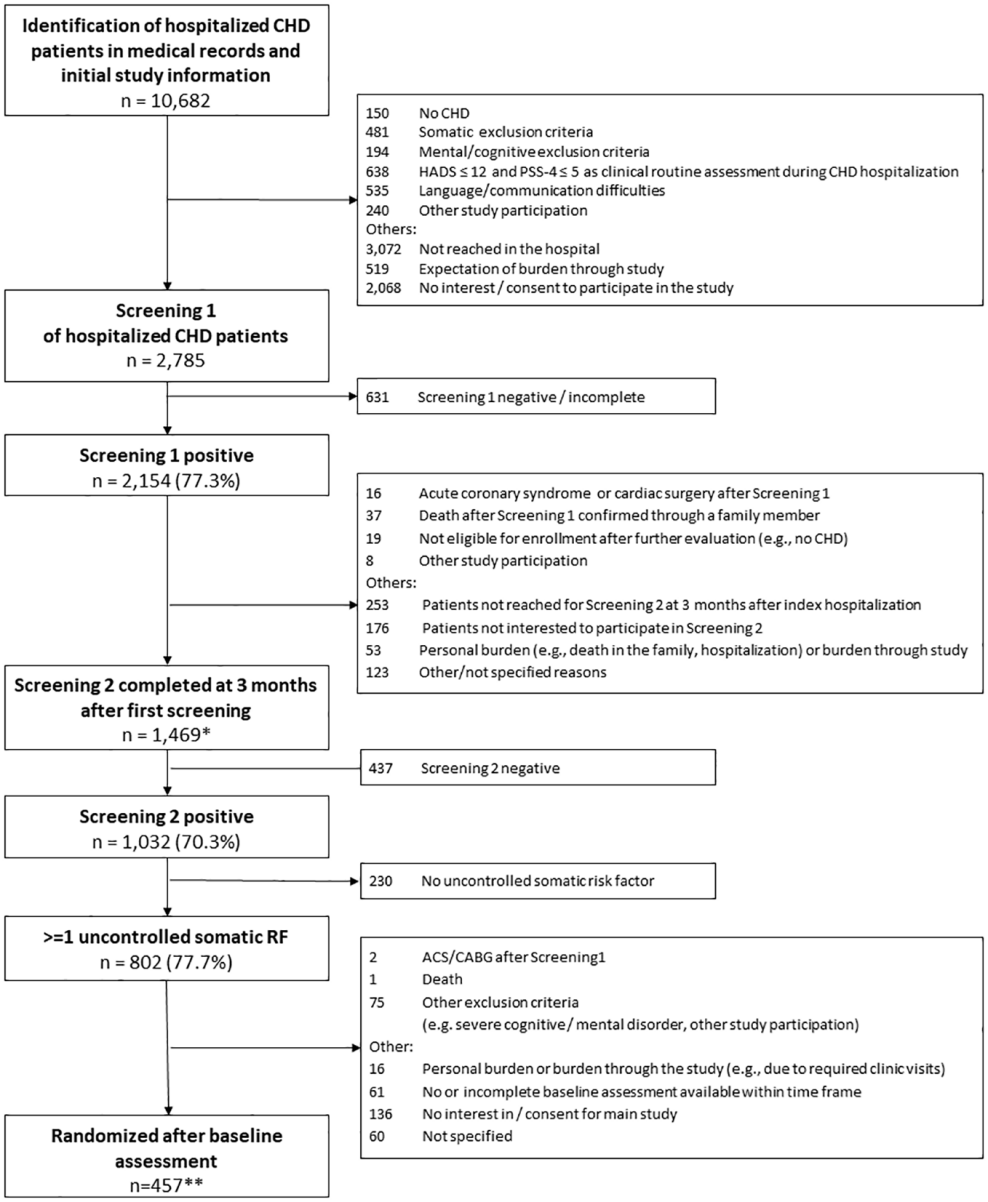
Flow chart of TEACH screening process. CHD, Coronary heart disease; HADS, Hospital Anxiety and Depression Scale; PSS-4 ,  4-item Perceived Stress Scale; RF, risk factor; ACS, Acute coronary syndrome; CABG, Coronary artery bypass graft surgery. *including 1 patient with negative initial distress screening; **including 6 patients with no documentation of an uncontrolled medical risk factor.

We identified 10,682 patients with a suspected diagnosis of CHD of whom 2,785 consented and completed the initial distress screening (Screening 1). Reasons for non-inclusion can be seen in **Figure 1**.

#### Screening 1

The patients completing the first screening were on average 65.0 (SD 10.1) years old and 78.4% were men. None of the patients identified their gender as diverse. Among the screened patients, 2,154 (77.3%) fulfilled the distress criterion. Of these, 58% showed elevated scores on both the HADS and PSS-4, while 31% of patients scored positive on the PSS-4 alone and 11% on the HADS alone.

Exclusion criteria discovered after the distress screening led to exclusion of 80 patients. All other patients were invited to the second, telephone-based screening assessment (Screening 2) three months later.

#### Screening 2

Of the 1,469 patients who participated in the second screening, 1,032 patients (70.3%) reported elevated HADS and/or PSS-4 scores. Reasons for not participating in the second screening can be seen in **Figure 1**.

In patients with a second positive distress screening, assessment of cardiac risk yielded 802 patients (77.7%) with one or more insufficiently controlled medical risk factors. Exclusion criteria were found at in-depth inquiry in 78 patients and logistic or patient-related reasons, including refusal of granting the second informed consent required for RCT participation left a final sample of 457 patients who were randomized. This number included one patient who was distressed at Screening 2 but not Screening 1 and 6 patients with no documented uncontrolled risk factor in Screening 2 who were erroneously included. These patients were left in the sample in order to comply with the intention to treat principle planned for the main analysis.

### Sex and age effects on screening outcome and enrollment

At all-time points, eligible women were significantly older than eligible men. The mean age difference ranged between 2.4 and 3.2 years (all p<0.001) in the different screening steps. Mean age recorded at initial screening slightly decreased in the remaining sample and both sexes over the screening process: It was 64.1 (SD 9.9) years in patients participating in the second screening and 62.6 (SD 9.5) years in the randomized subgroup. At the same time, the percentage of women in the sample slightly increased from 21.6% in those participating in the first screening to 23.0% in the randomized subgroup. This slight increase in women might be due to higher rates of distress in women vs. men at both, the first (85.4% vs. 75.1%; p<0.001) and second screening (75.5% vs. 68.7%; p=0.018).

In bivariate analyses, distressed patients with higher HADS or PSS-4 values at Screening 1 were more likely to screen positive at Screening 2 (p<0.001 for both scores). Independent effects of age, sex, HADS, and PSS-4 scores at first screening on dropout until second screening and randomization respectively were examined in multivariable logistic regression analyses. Older age was consistently related to dropout (**Table 1**), while sex had no independent effect. However, dropout until randomization was less frequent in patients with higher dimensional PSS-4 scores (p<0.001) and – on a trend level – also with higher HADS total scores (p=0.051).

**Table 1:** Multivariable baseline predictors of dropout before randomization among 2,785 patients with positive initial screening (logistic regression analysis).

	Dropout before screening 2	Dropout before randomization
	multivariable OR (95% CI)	multivariable OR (95% CI)
**Age**	**1.022 (1.013-1.031)**	**1.026 (1.015-1.037)**
Female sex	1.208 (0.977-1.495)	1.052 (0.818-1.355)
HADS total	1.001 (0.987-1.015)	0.984 (0.969-1.000)
**PSS-4**	1.017 (0.978-1.057)	**0.922 (0.883-0.964)**

OR, odds ratio; CI, confidence interval; HADS, Hospital Anxiety and Depression Scale; PSS-4 = 4-item Perceived Stress Scale. Significant predictors presented in boldface.

### Baseline characteristics of the entire randomized sample and by sex

At randomization, patients were on average 62.9 (SD 9.5) years old. The women were significantly older (by 2.3 years) than the men (p=0.025; see **Table 2**).

**Table 2:** Sociodemographic and medical baseline data of randomized patients.

	Total (n=457)	Men (n=352)	Women (n=105)	Signif.
	(Mean ± SD)	(Mean ± SD)	(Mean ± SD)	(p)
Age (years)	62.9 ± 9.5	62.4 ± 9.2	64.7 ± 10.2	**0.025**
BMI (kg/m²)	29.3 ± 5.5	29.4 ± 5.2	29.9 ± 6.6	0.487
SBP (mmHg)	129.6 ± 18.4	130.1 ± 18.6	128.0 ± 17.9	0.318
DBP (mmHg)	79.1 ± 11.3	79.9 ± 11.2	76.1 ± 11.1	**0.003**
Heart rate (1/min)	67.5 ± 11.4	67.7 ± 11.9	66.9 ± 9.3	0.481
# uncontrolled RFs (screening)	1.5 ± 0.7	1.4 ± 0.7	1.5 ± 0.7	0.286
Heart QoL total score	1.5 ± 0.7	1.6 ± 0.7	1.4 ± 0.6	**0.016**
Social demograhics	N (%)	N (%)	N(%)	
Partnership Living with partner Partner, living separately No partnership	317 (69.8) 41 (9.0) 96 (21.1)	264 (75.2) 30 (8.6) 57 (16.2)	53 (51.5) 11 (10.7) 39 (37.9)	**<0.001**
Highest school education Basic education (“Hauptschule”) Middle school (“Realschule”) (technical) baccalaureate (“Abitur”) Other/unknown/no response	172 (37.6) 118 (25.8) 155 (33.9) 12 (2.6)	126 (35.8) 87 (24.7) 129 (36.6) 10 (2.8)	46 (43.8) 31 (29.5) 26 (24.8) 2 (1.9)	0.125
Retired	191 (41.8)	136 (38.6)	55 (52.4)	**0.012**
Private health insurance	70 (15.3)	60 (17.0)	10 (9.5)	0.060
Risk factors	N (%)	N (%)	N (%)	
Currently smoking	79 (17.3)	63 (17.9)	16 (15.2)	0.853
Diabetes	148 (32.4)	109 (31.0)	39 (37.1)	0.381
HbA1c ≥ 7.0% ^§^	68 (15.3)	52 (15.1)	16 (16.0)	0.829
Hypertension	384 (84.0)	302 (85.8)	82 (78.1)	0.167
BP ≥ 140/90 mmHg ^$^	148 (32.5)	117 (33.3)	31 (29.5)	0.464
Dyslipidemia	384 (84.0)	296 (84.1)	88 (83.8)	0.649
LDL ≥ 70 mg/dl ^§^	165 (36.9)	121 (35.0)	44 (43.6)	0.115
Inactive (IPAQ) (n=449 ^&^	202 (45.0)	146 (41.8)	56 (56.0)	**0.012**
Family history of premature MI/stroke	153 (33.5)	105 (29.8)	48 (45.7)	**0.009**
Clinical severity	N (%)	N (%)	N (%)	
NYHA class 0-I	316 (69.1)	239 (67.9)	77 (73.3)	
II	88 (19.3)	66 (18.8)	22 (21.0)	0.099
III-IV	53 (11.6)	47 (13.4)	6 (5.7)	
CCS class I	274 (60.0)	220 (62.5)	54 (51.4)	
II	123 (26.9)	90 (25.6)	33 (31.4)	0.197
III-IV	56 (12.2)	40 (11.3)	16 (15.3)	
unknown	4 (0.9)	2 (0.6)	2 (1.9)	
Cardiac history	N (%)	N (%)	N (%)	
History of myocardial infarction	280 (61.3)	221 (62.8)	59 (56.2)	0.390
LVEF < 40% ^#^	66 (14.4)	55 (15.6)	11 (10.5)	0.314
Current/previous AFib	114 (24.9)	87 (24.7)	27 (25.7)	0.427
History of PCI	336 (73.5)	261 (74.1)	75 (71.4)	0.418
History of CABG	160 (35.0)	138 (39.2)	22 (21.0)	**<0.001**
ICD implanted	47 (10.3)	39 (11.1)	8 (7.6)	0.256
Cardiac rehabilitation in last year	229 (50.1)	184 (52.1)	45 (43.4)	0.115

BMI, Body mass index; SBP, systolic blood pressure; DBP, diastolic blood pressure; RF, risk factor; MI, myocardial infarction; NYHA, New York Heart Association functional class; CCS, Canadian Cardiovascular Society functional class; LVEF, left ventricular ejection fraction; AFib, atrial fibrillation; PCI, percutaneous coronary intervention; CABG, coronary artery bypass graft; ICD, implanted cardioverter-defibrillator. ^§^No blood samples available for 10 (LDL) or 13 (HbA1c) patients, respectively. ^§^No valid blood pressure measurement available for one patient. ^&^IPAQ incomplete in 8 patients. ^#^No current measurement of LVEF available in 23 patients. Significant p values are shown in boldface.

#### Sociodemographic variables

Substantial differences between men and women were seen in partnership and occupational status. Significantly more men than women lived with a partner (75.2% vs. 51.5%; p<0.001) and more women than men were already retired (52.4% vs. 38.6%; p=0.012). Men tended to have a higher rate of private health insurance – an indicator of higher socioeconomic status in Germany - than women (17.0% vs. 9.5%; p=0.06), while differences in educational level were not statistically significant.

#### Risk factors

Overall medical risk factor burden was unrelated to sex. The mean medical risk score of insufficiently controlled risk factors identified during the eligibility check in Screening 2 was 1.5. For details see **Table 2**.

Among the individual somatic or behavior-dependent risk factors, women were more likely than men to report physical inactivity on the IPAQ (56.0% vs. 41.8%; p=0.012) and a positive family history of premature cardiovascular disease (i.e. myocardial infarction or stroke occurring in a first-degree relative before age 55 in men or age 65 in women; 45.7% vs. 29.8%; p=0.009). On the other hand, men showed a significantly higher mean diastolic blood pressure than women (79.9 ± 11.2 vs. 76.1 ± 11.1 mmHg; p=0.003). However, the mean diastolic blood pressure was in the normal range for both sexes. No sex differences were observed for mean systolic blood pressure and heart rate, showing normal mean values around 130 mmHg and 67 bpm, respectively. Prevalences of other risk factors were unrelated to sex. Most frequent were dyslipidemia and hypertension (84% each). Hypertension was insufficiently controlled (≥ 140/90 mmHg) in 32.5% and LDL cholesterol was ≥ 70 mg/dl in 36.9%. Diabetes was known in 32.4% and 15.3% had an HbA1c of ≥ 7.0%. The mean BMI of 29.3 kg/m² indicated an overweight sample. More than one in six patients (17.3%) were current smokers, all without relevant sex differences (see **Table 2** for details).

#### Clinical characteristics

Clinical disease severity also did not differ by sex. It showed mainly no or asymptomatic heart failure (NYHA classes 0 to I: 69.1%) and no anginal symptoms during regular exercise (CCS class I: 60.0%). However, severe heart failure (NYHA III or IV) and severe angina (CCS III or IV) were present in approximately 12% each (**Table 2**).

Cardiac history only differed between sexes for coronary artery bypass surgery, which had been performed in 39.2% of the men but only 21.0% of the women (p<0.001). Of the whole sample, 61.3% had experienced a myocardial infarction and LVEF was below 40% in 14.4%. Almost 3 out of 4 patients had received a percutaneous coronary intervention and 10.3% were implanted with an internal defibrillator. Approximately 25% had a history of current or previously documented atrial fibrillation. Half of the patients had participated in cardiac rehabilitation during the preceding year, each with no significant sex difference.

#### Mental health

The most pronounced sex differences were seen with respect to mental health (**Table 3**). In the whole sample, 54% reported a physician-diagnosed mental disorder, most frequently a depressive disorder (40.3%). However, prevalences for most mental disorders were substantially higher in women than men (depressive disorders: 54.4% vs. 36.1%, p=0.002; anxiety disorders: 29.5% vs. 15.6%, p=0.005; somatoform disorders: 13.3% vs. 3.1%, p<0.001; any mental disorder: 71.4% vs. 48.9%, p < 0.001). Over 40% of patients had already received some mental health treatment, with women again showing a markedly higher rate than men (58.1% vs. 37.2%; p<0.001). The higher prevalence of diagnosed mental disorders in women was also reflected in their reduced quality of life (p=0.016; see **Table 2**) and the reported distress levels: Elevated HADS total scores >12 were found in 83.3% of women and 70.7% in men (p=0.008). Almost all women (97.1%) had elevated PSS-4 scores > 5, as compared to 89.2% of the men (p=0.012).

**Table 3:** Mental disorders, mental health treatments, and distress at baseline.

	Total (n=457)	Men (n=352)	Women (n=105)	Signif.
	N (%)	N (%)	N (%)	(p)
Known depression*	184 (40.3)	127 (36.1)	57 (54.3)	**0.002**
Known anxiety disorder*	86 (18.8)	55 (15.6)	31 (29.5)	**0.005**
Known somatoform disorder*	25 (5.5)	11 (3.1)	14 (13.3)	**<0.001**
Known abuse/addiction*	44 (9.6)	39 (11.1)	5 (4.8)	0.112
Other mental disorder*	54 (11.8)	42 (11.9)	12 (11.4)	0.765
Any mental disorder*	247 (54.0)	172 (48.9)	75 (71.4)	**<0.001**
History of mental health treatment	192 (42.0)	131 (37.2)	61 (58.1)	**<0.001**
HADS total > 12	337 (73.7)	249 (70.7)	88 (83.3)	**0.008**
PSS-4 > 5	416 (91.0)	314 (89.2)	102 (97.1)	**0.012**
PSS-4 > 5 and HADS total >12	323 (70.7)	236 (67.0)	87 (82.9)	**0.002**

*Self-report of physician-diagnosed disorder; multiple diagnoses per patient possible. Significant p values are shown in boldface.

#### Treatment satisfaction

**Figure 2:**
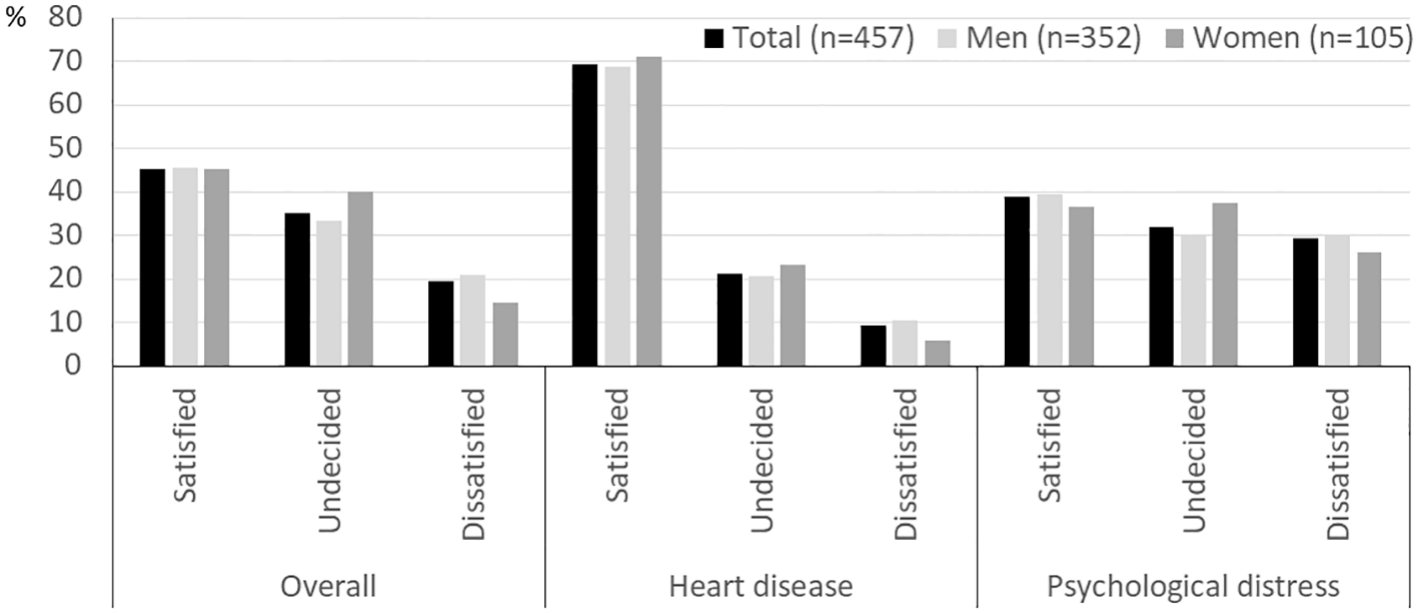
Satisfaction with routine treatment received before randomization by sex. Significant differences between satisfaction with care for heart disease vs. psychological distress, care for heart disease vs. overall care, and care for distress vs. overall care. Two highest and two lowest categories combined. All p<0.001. All sex effects not significant.

Satisfaction with medical treatment before enrollment in the RCT phase of the trial was rated separately for treatment for heart disease, treatment for psychological distress, and overall treatment. As shown in **Figure 2**, treatment satisfaction did not differ between sexes for any of these three dimensions (all p>0.2). However, substantial differences were seen between satisfaction with treatment for the different conditions. Less than half of the patients (45.4%) were rather or completely satisfied with their overall treatment. This rate was markedly higher for satisfaction with cardiac treatment (69.3%) but even lower for psychosocial care (38.8%). All differences were highly significant at p < 0.001.

## Discussion

TEACH is the first confirmatory multicenter trial of a BCC intervention in a European health care system and the first BCC trial in a uniform CHD sample. It succeeded in enrolling a typical CHD patient cohort of respectable size from seven sites across Germany. Besides providing valuable information about the practicability and results of the TEACH screening algorithm, the current analyses yield important information on relevant baseline characteristics of the TEACH cohort which may inform the upcoming longitudinal analyses. In addition, future studies on BCC or other psychosocial/behavioral interventions in cardiac patients may build on our results to fine-tune their interventions to the baseline characteristics and resulting needs of distressed CHD patients in general and, in particular, the different problem areas of men and women.

### Screening process

Initial distress observed in the hospital may remit either spontaneously with good medical care, return to the familiar home environment and/or as a result of (multimodal) cardiac rehabilitation. However, our data show that over 70% of initially distressed CHD patients who were available for our second screening 3 months later were still (or again) distressed at that time and were likely to benefit from psychosocial support. At the same time, more than 3 out of 4 still distressed CHD patients showed insufficient medical risk factor control, indicating an additional need for risk factor counseling or medication adjustment in these patients.

As the TEACH BCC intervention mainly addresses psychological distress and cardiovascular risk factors, the applied screening algorithm appears to facilitate the selection of patients for whom such a BCC intervention may be particularly beneficial.

Our stepwise screening algorithm allowed us to systematically analyze how age, sex, and initial distress predict dropout before randomization into the RCT. Interestingly, although men were overrepresented in our initial screening sample, due to a generally higher percentage of men in typical hospitalized CHD patient populations from which our sample was drawn, female CHD patients were no less likely to stay in the study throughout the screening process and until randomization than men. In the final randomized sample, the underrepresentation of women was even slightly diminished (from 21.6% to 23.0%). Sex was, however, not an independent predictor of dropout.

In contrast, older age was clearly predictive of early dropout. Women were on average 2-3 years older than men throughout the screening process, which is in line with the later onset of CHD in women than in men. The disadvantage of older age for retention of women in the study may have been counterbalanced by the fact that women showed consistently higher levels of distress than men. This difference persisted into the RCT cohort, even after preselecting only patients with at least mild distress at two time points. Elevated absolute levels of distress were, in turn, associated with a higher likelihood of participating in the RCT which may have offset the adverse effect of higher age in the women.

The positive effect of higher reported distress on successful RCT enrollment may be due to the simple fact that within the distressed patients higher initial distress increased the risk for a positive second screening result. However, even in patients with a positive second screening result, a higher HADS score at first screening still tended to predict randomization (p=0.076; data not shown). Therefore, the higher participation rate of more distressed patients in the RCT may indicate the patients’ wish for potentially receiving the study intervention to improve their health care. This assumption is supported by the relatively poor rating of satisfaction with health care for the time period before RCT enrollment, especially in the psychosocial domain. The imbalance between satisfaction with somatic and psychosocial care is striking and calls for improving psychosocial health care for CHD patients, which is actually mandated by the German Disease Management Program for chronic CHD (27) but apparently insufficiently implemented. On the other hand, other patients dropped out because they perceived study participation as too burdensome, given their already demanding life situation.

### Baseline characteristics and sex differences in the RCT cohort

In our sample, women were older than men, far less likely to live with a partner, more likely to be retired and physically inactive and had much lower rates of surgical revascularizations than men, confirming previously reported inequalities in medical CHD treatments (28). They reported higher rates of a positive family history of premature atherosclerotic disease, previously reported to increase the risk for mental distress in cardiac patients (29) and were much more affected by psychological distress and diagnosed mental disorders than men. Accordingly, they had received more mental health care before study enrollment than men, which was, however, not associated with better satisfaction with that care.

In summary, while disease severity, cumulative medical risk factor burden, and most aspects of cardiac history were comparable between men and women, we found sex-specific differences in care needs. For example, women were much more likely than men to live alone and be retired, possibly requiring interventions to overcome loneliness, together with specific treatments of their higher rates of mental disorders.

As reported in earlier psychosocial intervention trials, such as SWITCHD (6), SUPRIM (7), MHART (15) or ENRICHD (14), women may need different interventions than men, with more need for empathic listening and self-assertiveness training in women and a stronger focus on risk factor education, anger control and practical guidance for men. BCC offers the opportunity to address sex and other individual differences by taking into account individual patient preferences and treatment demands. Nevertheless, raising awareness among care managers of typical sex and gender differences in treatment needs may help them to specifically enquire for such issues during individual intervention planning and delivery.

Another relevant information for intervention design may be the relative frequency of insufficiently controlled medical risk factors. Our study participants suffered most frequently from physical inactivity, although half of them had participated in cardiac rehabilitation during the preceding year. Over 30% had insufficiently controlled blood pressure and elevated LDL cholesterol, and a good 15% had elevated HbA1c or were still smoking, respectively. Beyond the high level of mental distress and disorders, these uncontrolled medical risk factors therefore deserve particular attention during the intervention.

When comparing the TEACH cohort to patient cohorts from previous (blended) collaborative care (CC) trials in patients with heart disease [e.g., TEAMCare (9), Bypassing the Blues (30), Hopeful Heart (31), COPES (16), MOSAIC (32), COMPASS (10)], we observed a much more equal sex distribution in all previous (B)CC trials from the USA. However, as shown, an unequal sex distribution was already seen in the first TEACH screening assessment and the percentage of women even slightly increased during the screening process, thus arguing against a sex or gender bias in patient enrollment for TEACH which could also not be found in our multivariable regression analysis. The sex ratio in TEACH is rather in line with the sex ratio observed in hospitalized German CHD patients aged 18-85 overall (33) and in international trials of cardiac interventions in CHD patients, where men are almost always overrepresented, as long as enrollment is not specifically tied to sex. For example, in ORBITA-2 (34), the POST-PCI trial (35), and ISCHEMIA (36) studying a total of >7,000 CHD patients, men made up between 77% and almost 80% of participants. This suggests that the 77% men seen in TEACH are quite typical for CHD patients in general hospitals and those participating in intervention trials in cardiology.

The percentages of men seen in TEACH were also similar to those in other psychological intervention trials with CHD patient samples from Europe [e.g., (7, 13).

The difference of the sex ratios in previous (B)CC studies vs. cardiology studies (and psychological intervention studies from Europe, including TEACH) may in part be due to the well-known higher rates of depression and distress in women vs. men who may therefore be more likely to qualify for depression treatment studies. However, the main reason for the difference will likely be the requirement of American funding agencies to enroll similar numbers of men and women in psychosocial/behavioral intervention trials.

Beyond the higher portion of women, differences in other patient characteristics between the TEACH sample and previous (B)CC intervention trials are important for future comparisons of intervention effects among the trials, because different baseline characteristics may have an impact on treatment goals and effectiveness. For example, the TEAMCare study (9), the only BCC study so far that showed improvements in both, mental well-being and medical risk factors, enrolled patients from a primary care setting with diabetes and/or CHD, which was, however, only documented in < 30%. Accordingly, their patients had much higher rates of diabetes with higher mean BMI and HbA1c and were substantially younger than the TEACH cohort. Their LDL cholesterol at baseline was also much higher than in TEACH, probably reflecting less strict guideline recommendations in vigor at that time.

The only other large randomized BCC trial in cardiac patients, the Hopeful Heart trial (31), randomized depressed patients with systolic heart failure of any origin who cannot easily be compared with the TEACH CHD cohort. However, the mean age in the randomized group was comparable to TEACH, as were rates of hypertension and smoking.

The TEACH sample is more similar to the post-coronary bypass surgery sample of depressed CHD patients from the Bypassing the Blues trial (30) who were slightly older than the patients in TEACH but had a similar risk factor profile.

All of the studies mentioned above used elevated depressive symptoms as an inclusion criterion. However, since accumulating evidence suggests that not only depression but rather any type of distress may impair prognosis in CHD patients, TEACH chose to apply a broader psychological inclusion criterion of at least mild distress on the HADS and/or elevated perceived stress on the PSS-4. Both, elevated HADS scores [e.g., (37, 38)] and elevated PSS-4 scores (25) have been shown to impair prognosis in CHD patients. Given the broader psychological inclusion criterion, higher percentages of patients screened positive in the initial TEACH screening step than in the previous BCC studies. On the other hand, TEACH aimed to only include patients in whom distress persisted over at least 3 months, in order to exclude patients with spontaneous remission of distress after returning home from the hospital (and possibly subsequent cardiac rehabilitation). Here it is remarkable that 7 out of 10 patients with initial distress were still (or again) distressed three months later.

A similar two-step screening procedure has been applied in the COPES trial (16). In that trial, almost 50% of patients after an acute coronary syndrome who were initially depressed lay below the cut-off at three months.

Using broader inclusion criteria, Huffman et al. (32) randomized 183 anxious or depressed patients who had suffered a recent cardiac event into the MOSAIC trial of a 24-week multicomponent CC intervention. Patients in that trial showed a similar risk factor profile and percentage of previously known depression as the TEACH sample.

### Strengths

TEACH is the first confirmatory multicenter efficacy trial of a BCC intervention in a European health care system and the first BCC trial in a uniform CHD sample. Its systematic screening algorithm, considering both, distress and insufficiently controlled risk factors persisting beyond the acute treatment (and early rehabilitation) phase at the same time, identified a high-risk patient group particularly suited for a BCC intervention. It also allowed to identify factors associated with dropout during the screening process and showed relevant sex differences in psychological and medical burden that can inform future BCC trials.

### Limitations

The current analyses have a number of limitations. First, TEACH was conducted in a mainly white German population with sufficient German language skills and its results cannot be generalized to non-white and migrant samples or different cultural backgrounds and health care systems. Reasons for non-inclusion in the initial screening step could only be ascertained rather globally. Since patients had not consented to study participation, for legal reasons no identifying information could be used. Thus, sex, age, and disease-related factors may have biased initial willingness to participate in an unknown way. For patients participating in the initial screening, dropout criteria could not always be documented, leaving some uncertainty. This includes the unknown impact of the Covid-19 pandemic on the enrollment process which started in the summer of 2020. Possible effects of the pandemic on sample composition could not be systematically assessed and may have introduced some bias as during the first waves of the pandemic fewer patients were treated on the cardiology wards than in previous years and some patients refused to attend in person for trial-related assessments out of fear of contracting an infection.

Finally, the baseline data do not provide information about the efficacy of the BCC intervention for which data will be available after obtaining and cleaning the data from the follow-up assessments of up to 30 months after randomization.

## Conclusion

The TEACH screening algorithm proved to be effective in identifying persistently distressed patients with insufficiently controlled medical risk factors. As in typical medical intervention studies in CHD patients, the randomized sample is predominantly male and showed a typical risk factor profile. However, the percentage of women in TEACH is substantially lower than in previous (B)CC studies with cardiac patients from the USA that obviously oversampled female participants. Sex was, however, not independently affecting enrollment in TEACH between first screening and randomization. In the randomized sample, however, there were substantial sex differences in sociodemographic data, mental health and some aspects of cardiovascular risk and treatment, which should be considered in future analyses of the TEACH data set and in planning future (B)CC interventions. The low patient satisfaction with routine psychosocial care calls for effective interventions to overcome this deficit.

## Data Availability

The raw data supporting the conclusions of this article will be made available by the authors, without undue reservation.

## References

[1] Visseren FLJ Mach F Smulders YM Carballo D Koskinas KC Bäck M. 2021 ESC Guidelines on cardiovascular disease prevention in clinical practice. Eur Heart J. (2021) 42:3227–337. doi: 10.1093/eurheartj/ehab484 34458905

[2] Chen HY Saczynski JS Lapane KL Kiefe CI Goldberg RJ. Adherence to evidence-based secondary prevention pharmacotherapy in patients after an acute coronary syndrome: A systematic review. Heart Lung. (2015) 44:299–308. doi: 10.1016/j.hrtlng.2015.02.004 25766041 PMC8075173

[3] Berkman LF Blumenthal J Burg M Carney RM Catellier D Cowan MJ. Effects of treating depression and low perceived social support on clinical events after myocardial infarction: the Enhancing Recovery in Coronary Heart Disease Patients (ENRICHD) Randomized Trial. JAMA. (2003) 289:3106–16. doi: 10.1001/jama.289.23.3106 12813116

[4] Glassman AH O'Connor CM Califf RM Swedberg K Schwartz P Bigger JT Jr. Sertraline treatment of major depression in patients with acute MI or unstable angina. JAMA. (2002) 288:701–9. doi: 10.1001/jama.288.6.701 12169073

[5] Kim JM Stewart R Lee YS Lee HJ Kim MC Kim JW. Effect of escitalopram vs placebo treatment for depression on long-term cardiac outcomes in patients with acute coronary syndrome: A randomized clinical trial. JAMA. (2018) 320:350–8. doi: 10.1001/jama.2018.9422 PMC6583706 30043065

[6] Orth-Gomér K Schneiderman N Wang HX Walldin C Blom M Jernberg T. Stress reduction prolongs life in women with coronary disease: the Stockholm Women's Intervention Trial for Coronary Heart Disease (SWITCHD). Circ Cardiovasc Qual Outcomes. (2009) 2:25–32. doi: 10.1161/CIRCOUTCOMES.108.812859 20031809

[7] Gulliksson M Burell G Vessby B Lundin L Toss H Svärdsudd K. Randomized controlled trial of cognitive behavioral therapy vs standard treatment to prevent recurrent cardiovascular events in patients with coronary heart disease: Secondary Prevention in Uppsala Primary Health Care project (SUPRIM). Arch Intern Med. (2011) 171:134–40. doi: 10.1001/archinternmed.2010.510 21263103

[8] Li T Jiang H Ding J. The role of exercise-based cardiac rehabilitation after percutaneous coronary intervention in patients with coronary artery disease: a meta-analysis of randomised controlled trials. Acta Cardiol. (2024) 79:127–35. doi: 10.1080/00015385.2023.2266650 38465795

[9] Katon WJ Lin EH Von Korff M Ciechanowski P Ludman EJ Young B. Collaborative care for patients with depression and chronic illnesses. N Engl J Med. (2010) 363:2611–20. doi: 10.1056/NEJMoa1003955 PMC3312811 21190455

[10] Rossom RC Solberg LI Magnan S Crain AL Beck A Coleman KJ. Impact of a national collaborative care initiative for patients with depression and diabetes or cardiovascular disease. Gen Hosp Psychiatry. (2017) 44:77–85. doi: 10.1016/j.genhosppsych.2016.05.006 27558106

[11] Bosselmann L Fangauf SV Herbeck Belnap B Chavanon ML Nagel J Neitzel C. Blended collaborative care in the secondary prevention of coronary heart disease improves risk factor control: Results of a randomised feasibility study. Eur J Cardiovasc Nurs. (2020) 19:134–41. doi: 10.1177/1474515119880062 31564125

[12] Herrmann-Lingen C Albus C de Zwaan M Geiser F Heinemann K Hellmich M. Efficacy of team-based collaborative care for distressed patients in secondary prevention of chronic coronary heart disease (TEACH): study protocol of a multicenter randomized controlled trial. BMC Cardiovasc Disord. (2020) 20:520. doi: 10.1186/s12872-020-01810-9 33302871 PMC7731481

[13] Deter HC Weber C Herrmann-Lingen C Albus C Juenger J Ladwig KH. Gender differences in psychosocial outcomes of psychotherapy trial in patients with depression and coronary artery disease. J Psychosom Res. (2018) 113:89–99. doi: 10.1016/j.jpsychores.2018.08.005 30190055

[14] Schneiderman N Saab PG Catellier DJ Powell LH DeBusk RF Williams RB. Psychosocial treatment within sex by ethnicity subgroups in the Enhancing Recovery in Coronary Heart Disease clinical trial. Psychosom Med. (2004) 66:475–83. doi: 10.1097/01.psy.0000133217.96180.e8 15272091

[15] Cossette S Frasure-Smith N Lespérance F. Nursing approaches to reducing psychological distress in men and women recovering from myocardial infarction. Int J Nurs Stud. (2002) 39:479–94. doi: 10.1016/s0020-7489(01)00051-7 11996869

[16] Davidson KW Rieckmann N Clemow L Schwartz JE Shimbo D Medina V. Enhanced depression care for patients with acute coronary syndrome and persistent depressive symptoms: coronary psychosocial evaluation studies randomized controlled trial. Arch Intern Med. (2010) 170:600–8. doi: 10.1001/archinternmed.2010.29 PMC2882253 20386003

[17] Zigmond AS Snaith RP. The hospital anxiety and depression scale. Acta Psychiatr Scand. (1983) 67:361–70. doi: 10.1111/j.1600-0447.1983.tb09716.x 6880820

[18] Herrmann-Lingen C Buss U Snaith RP. HADS-D. Hospital anxiety and depression scale—Deutsche Version. 4th. Snaith vRP Zigmond AS, editors. Bern: Hogrefe: Deutsche Adaptation der Hospital Anxiety and Depression Scale (HADS (2018). und.

[19] Warttig SL Forshaw MJ South J White AK. New, normative, English-sample data for the Short Form Perceived Stress Scale (PSS-4). J Health Psychol. (2013) 18:1617–28. doi: 10.1177/1359105313508346 24155195

[20] Piepoli MF Hoes AW Agewall S Albus C Brotons C Catapano AL. 2016 European Guidelines on cardiovascular disease prevention in clinical practice: The Sixth Joint Task Force of the European Society of Cardiology and Other Societies on Cardiovascular Disease Prevention in Clinical Practice (constituted by representatives of 10 societies and by invited experts)Developed with the special contribution of the European Association for Cardiovascular Prevention & Rehabilitation (EACPR). Eur Heart J. (2016) 37:2315–81. doi: 10.1093/eurheartj/ehw106 PMC4986030 27222591

[21] Khaw KT Wareham N Bingham S Welch A Luben R Day N. Combined impact of health behaviours and mortality in men and women: the EPIC-Norfolk prospective population study. PloS Med. (2008) 5:e12. doi: 10.1371/journal.pmed.0050012 18184033 PMC2174962

[22] Bjelland I Dahl AA Haug TT Neckelmann D. The validity of the Hospital Anxiety and Depression Scale. An updated literature review. J Psychosom Res. (2002) 52:69–77. doi: 10.1016/s0022-3999(01)00296-3 11832252

[23] Klein EM Brähler E Dreier M Reinecke L Müller KW Schmutzer G. The German version of the Perceived Stress Scale - psychometric characteristics in a representative German community sample. BMC Psychiatry. (2016) 16:159. doi: 10.1186/s12888-016-0875-9 27216151 PMC4877813

[24] Ezzati A Jiang J Katz MJ Sliwinski MJ Zimmerman ME Lipton RB. Validation of the Perceived Stress Scale in a community sample of older adults. Int J Geriatr Psychiatry. (2014) 29:645–52. doi: 10.1002/gps.4049 PMC4013212 24302253

[25] Arnold SV Smolderen KG Buchanan DM Li Y Spertus JA. Perceived stress in myocardial infarction: long-term mortality and health status outcomes. J Am Coll Cardiol. (2012) 60:1756–63. doi: 10.1016/j.jacc.2012.06.044 PMC3601381 23040574

[26] Craig CL Marshall AL Sjöström M Bauman AE Booth ML Ainsworth BE. International physical activity questionnaire: 12-country reliability and validity. Med Sci Sports Exerc. (2003) 35:1381–95. doi: 10.1249/01.MSS.0000078924.61453.FB 12900694

[27] Gemeinsamer Bundesausschuss. DMP-Anforderungen-Richtlinie. Available online at: https://www.g-ba.de/downloads/62-492-3454/DMP-A-RL_2023-11-16_iK-2024-07-01.pdf (Accessed August 13, 2024).

[28] Fogg AJ Welsh J Banks E Abhayaratna W Korda RJ. Variation in cardiovascular disease care: an Australian cohort study on sex differences in receipt of coronary procedures. BMJ Open. (2019) 9:e026507. doi: 10.1136/bmjopen-2018-026507 PMC6661610 31337660

[29] Nagel J Chavanon ML Binder L Pieper A Wachter R Edelmann F. How family history of premature myocardial infarction affects patients at cardiovascular risk. Health Psychol. (2021) 40:754–63. doi: 10.1037/hea0001128 34914481

[30] Rollman BL Belnap BH LeMenager MS Mazumdar S Houck PR Counihan PJ. Telephone-delivered collaborative care for treating post-CABG depression: a randomized controlled trial. JAMA. (2009) 302:2095–103. doi: 10.1001/jama.2009.1670 PMC3010227 19918088

[31] Rollman BL Anderson AM Rothenberger SD Abebe KZ Ramani R Muldoon MF. Efficacy of blended collaborative care for patients with heart failure and comorbid depression: A randomized clinical trial. JAMA Intern Med. (2021) 181:1369–80. doi: 10.1001/jamainternmed.2021.4978 PMC8406216 34459842

[32] Huffman JC Mastromauro CA Beach SR Celano CM DuBois CM Healy BC. Collaborative care for depression and anxiety disorders in patients with recent cardiac events: the Management of Sadness and Anxiety in Cardiology (MOSAIC) randomized clinical trial. JAMA Intern Med. (2014) 174:927–35. doi: 10.1001/jamainternmed.2014.739 24733277

[33] Destatis. Statistischer Bericht - Diagnosen der Krankenhauspatienten (2022). Available online at: https://www.destatis.de/DE/Themen/Gesellschaft-Umwelt/Gesundheit/Krankenhaeuser/Publikationen/Downloads-Krankenhaeuser/statistischer-bericht-diagnosedaten-5231301227015.xlsx?blob=publicationFile (Accessed 2024/08/13).

[34] Rajkumar CA Foley MJ Ahmed-Jushuf F Nowbar AN Simader FA Davies JR. A placebo-controlled trial of percutaneous coronary intervention for stable angina. N Engl J Med. (2023) 389:2319–30. doi: 10.1056/NEJMoa2310610 PMC7615400 38015442

[35] Lee J Kang DY Kim H Choi Y Jo S Ahn JM. Routine stress testing after PCI in patients with and without acute coronary syndrome: A secondary analysis of the POST-PCI randomized clinical trial. JAMA Cardiol. (2024) 26:e241556. doi: 10.1001/jamacardio.2024.1556 PMC11209198 38922632

[36] Gaudino M Alexander JH Sandner S Harik L Kim J Stone GW. Outcomes by sex in the International Study of Comparative Health Effectiveness With Medical and Invasive Approaches (ISCHEMIA) trial. EuroIntervention. (2024) 20:551–60. doi: 10.4244/EIJ-D-24-00011 PMC11067519 38444364

[37] Meijer A Conradi HJ Bos EH Anselmino M Carney RM Denollet J. Adjusted prognostic association of depression following myocardial infarction with mortality and cardiovascular events: individual patient data meta-analysis. Br J Psychiatry. (2013) 203:90–102. doi: 10.1192/bjp.bp.112.111195 23908341

[38] Damen NL Versteeg H Boersma E Serruys PW van Geuns RJ Denollet J. Depression is independently associated with 7-year mortality in patients treated with percutaneous coronary intervention: results from the RESEARCH registry. Int J Cardiol. (2013) 167:2496–501. doi: 10.1016/j.ijcard.2012.04.028 22560933

