# Supplementary material for: Identification and characteristics of distressed patients with coronary heart disease and insufficiently controlled medical risk factors: baseline findings and sex differences from the multicenter TEACH trial
Source: Front Psychiatry. 2025 Jan 31;16:1494839. doi: 10.3389/fpsyt.2025.1494839 (PMC11825747; doi:10.3389/fpsyt.2025.1494839)
Supplement: Supplementary file 1 [file Table1.docx]

**Online supplement**

**Supplementary Table 1.** Inclusion and exclusion criteria for TEACH intervention trial.

***Inclusion criteria for Screening 1 and Screening 2***

1. Patients of any gender

2. Patient aged 18–85 years

3. Hospitalized in Department of Cardiology or Cardiac Surgery with a CHD diagnosis documented as:

a. coronary angiography (> 50% stenosis in ≥ 1 major coronary vessel)

b. and/or confirmed acute coronary syndrome

c. and/or history of coronary revascularization (percutaneous intervention or coronary artery bypass surgery)

4. Ability to speak, read and understand German

5. HADS > 12 and/or PSS-4 > 5

6. Written informed consent for screening procedure

***Inclusion criteria for main TEACH intervention trial (RCT)***

1. HADS > 12 and/or score on PSS-4 > 5

2. > = 1 insufficiently controlled cardiac risk factor as defined as:

a. Hypertension with blood pressure > = 140/90 mmHg

b. Hyperlipidemia with LDL-cholesterol > = 70 mg/dl

c. Current smoking

d. Diabetes with HbA1c > = 7.0%

e. Physical inactivity: self-report of < 150 min. of moderate or < 75 min. of vigorous physical activity per week

3. Written informed consent for study participation in main trial

***Exclusion criteria***

1. Severe cognitive impairment, defined as known dementia or inability to follow the assessment instructions

2. Communication difficulties (e.g. hard of hearing, aphasia)

3. Acute or severely disabling non-cardiac disease with estimated survival < 1 year

4. Need for more specialized cardiac or mental health interventions or structured rehabilitation programs:

a. recurrent ACS or coronary surgery after the index hospitalization

b. severe mental disorders (e.g. acute psychoses) or addiction (except tobacco)

5. Participation in another treatment trial likely to affect the outcomes of interest or interfering with the trial procedures
